# Delayed surgery versus nonoperative treatment for hip fractures in post-COVID-19 arena: a retrospective study of 145 patients

**DOI:** 10.1080/17453674.2020.1816617

**Published:** 2020-09-08

**Authors:** Bobin Mi, Lang Chen, Dake Tong, Adriana C Panayi, Fang Ji, Junfei Guo, Zhiyong Ou, Yingze Zhang, Yuan Xiong, Guohui Liu

**Affiliations:** a Department of Orthopedics, Union Hospital, Tongji Medical College, Huazhong University of Science and Technology, Wuhan, China;; b Department of Plastic Surgery, Brigham and Women’s Hospital, Harvard Medical College, Boston, USA;; c Department of Orthopedics, Shanghai Ninth People’s Hospital, Shanghai Jiaotong University School of Medicine, Shanghai, China;; d Department of Orthopedics, Changhai Hospital, Shanghai, China;; e Department of Orthopaedic Surgery, Third Hospital of Hebei Medical University, Shijiazhuang, China

## Abstract

Background and purpose — Following the outbreak of COVID-19 in December 2019, in China, many hip fracture patients were unable to gain timely admission and surgery. We assessed whether delayed surgery improves hip joint function and reduces major complications better than nonoperative therapy.

Patients and methods — In this retrospective observational study, we collected data from 24 different hospitals from January 1, 2020, to July 20, 2020. 145 patients with hip fractures aged 65 years or older were eligible. Clinical data was extracted from electronic medical records. The primary outcomes were visual analogue scale (VAS) score and Harris Hip Score. Major complications, including deep venous thrombosis (DVT) and pneumonia within 1 month and 3 months, were collected for further analysis.

Results — Of the 145 hip fracture patients 108 (median age 72; 70 females) received delayed surgery and 37 (median age 74; 20 females) received nonoperative therapy. The median time from hip fracture injury to surgery was 33 days (IQR 24–48) in the delayed surgery group. Hypertension, in about half of the patients in both groups, and cerebral infarction, in around a quarter of patients in both groups, were the most common comorbidities. Both VAS score and Harris Hip Score were superior in the delayed surgery group. At the 3-month follow-up, the median VAS score was 1 in the delayed surgery group and 2.5 in the nonoperative group (p < 0.001). Also, the percentage of complications was higher in the nonoperative group (p = 0.004 for DVT, p < 0.001 for pulmonary infection).

Interpretation — In hip fracture patients, delayed surgery compared with nonoperative therapy significantly improved hip function and reduced various major complications.

The incidence of hip fractures is on the rise, owing in part to the aging population, with the number of annual hip fractures worldwide expected to exceed 6 million by 2050 (Papadimitriou et al. 2017).

Nonoperative treatment of hip fractures requires long-term recovery, which increases the risk of complications such as pulmonary infection, pressure ulcers, urinary tract infection, and lower limb venous thrombosis, consequently leading to high mortality (Sinvani et al. 2020). Surgical treatment, hence, tends to be the preferable treatment for hip fracture patients. In terms of surgery, operation promptness is crucial for better clinical outcomes in the elderly (Ekeloef et al. 2019).

Delayed surgery may occur for various reasons, including delayed admission, or surgery in high-risk patients on certain medications or with comorbidities. Folowing the outbreak of COVID-19 in December 2019, in China, and particularly in the city of Wuhan, many hip fracture patients were unable to gain timely admission and surgery. Many factors contributed to this delay, including shortage of medical resources preventing hospitals from treating non-COVID-19 patients. In addition, fear of the high infection and mortality of SARS-CoV-2 deterred many patients from attending hospitals. As China is now in the post-outbreak period, many hip fracture patients are beginning to crowd into hospitals seeking surgical treatment.

In this retrospective study, we compared the outcomes of delayed surgery and nonoperative therapy in hip fracture patients. The clinical characteristics and surgical outcomes, including complications, were recorded.

## ^Patients and methods^

### ^Study design and participants^

This was a multicenter retrospective clinical study of delayed surgery versus nonoperative therapy in hip fracture patients during the COVID-19 pandemic. The study included 24 cohorts of adult inpatients (> 65 years old) from Wuhan Union Hospital, the Third Hospital of Hebei Medical University, Zhengzhou Orthopedic Hospital, Zhengzhou Central Hospital, Renhe Hospital of China Three Gorges University, Shanghai Changhai Hospital, People’s Hospital of Xiangzhou District, Xiangyang Central Hospital, People’s Hospital of Xiantao City, Qianjiang Hospital of Traditional Chinese Medicine, Taihe Hospital of Shiyan City, Nanchang Hospital of Traditional Chinese Medicine, Jiangxia People’s Hospital, Tianmen People’s Hospital, Shanghai Tenth People’s Hospital, People’s Hospital of Wuhan University, Wuhan Tongji Hospital, People’s Hospital of Shiyan City, Wuhan General Hospital of Guangzhou Military Command, Guangshui Hospital of Traditional Chinese Medicine, Guangshui Second People’s Hospital, People’s Hospital of Chibi City, Dongxi Lake People’s Hospital, and Badong People’s Hospital. The analyzed period spanned January 1, 2020 to July 20, 2020. All patients were diagnosed with hip fracture by orthopedic doctors on the basis of radiographies or CT scans. Surgeries were postponed due to the COVID-19 pandemic. Patients were included if they met the following criteria: at least 65 years old, admitted between January 1, 2020 and July 20, 2020, had a hip fracture that had not undergone surgery for more than 21 days. Patients with prior hip operation history were excluded. COVID-19 was diagnosed in accordance with the COVID-19 was diagnosed in accordance with the New Coronavirus Pneumonia Prevention and Control Program (6th edition) published by the National Health Commission of China (NHCC).

### ^Data collection^

Using a customized data collection form, we tabulated clinical characteristics, comorbidities, prior history, treatment, laboratory test results, and function scores, as provided by the 24 hospitals. All inpatients and family members received pulmonary CT, SARS-CoV-2 nucleic acid testing, and antibody testing prior to admission. Laboratory test results are the final results obtained prior to discharge. VAS score and Harris hip function score were graded by an orthopedic expert panel after 1 month and 3 months’ follow-up.

### ^Statistics^

Primary data were analyzed with SPSS software (version 23.0; IBM Corp, Armonk, NY, USA). Categorical variables were presented as frequency and percentages, while median and interquartile rage (IQR) values were used to describe continuous variables. A chi-square test was used to compare categorical variables between delayed surgery and nonoperative therapy; Fisher’s exact test was used when data were limited. A Kolmogorov–Smirnov test was performed to test whether continuous variables complied with normal distribution. An independent group t-test was applied to normally distributed continuous variables, otherwise the Mann–Whitney U-test was used. We defined excellent, good, not bad, and poor as Harris score 86–100, 71–85, 56–70, and 0–55 respectively, and the specific number and percentage in each group were calculated. P-values < 0.05 were considered statistically significant.

### ^Endpoints^

Due to the urgency and importance of the COVID-19 pandemic, we collected and analyzed the follow-up results of 145 elderly hip fracture patients as of July 20, 2020. Among them, the range of day from admission to the endpoint was 99 to 187 days. Kaplan–Meier curves were constructed to compare surgery and nonoperative therapy. 1-month and 3-month follow-up results were based on the outpatient clinical system and direct contact with patients or their family member.

### ^Ethics, funding, and potential conflicts of interest^

The study was approved by the Institutional Review Board of Union Hospital, Tongji Medical College, Huazhong University of Science and Technology and the requirement for consent was waived because of the retrospective analysis (IRB no. 2020-01-27). This study was supported by the National Key Research & Development Program of China (Grant Nos. 2018YFB2001502 and 2018YFB1105705), National Science Foundation of China (Grant No. 81772345), the National Health Commission of the People’s Republic of China (Grant Nos. ZX-01-018 and ZX-01-C2016153), and the Health Commission of Hubei Province (Grant No. WJ2019Z009). The authors report no conflicts of interest.

## ^Results^

**Figure 1.**
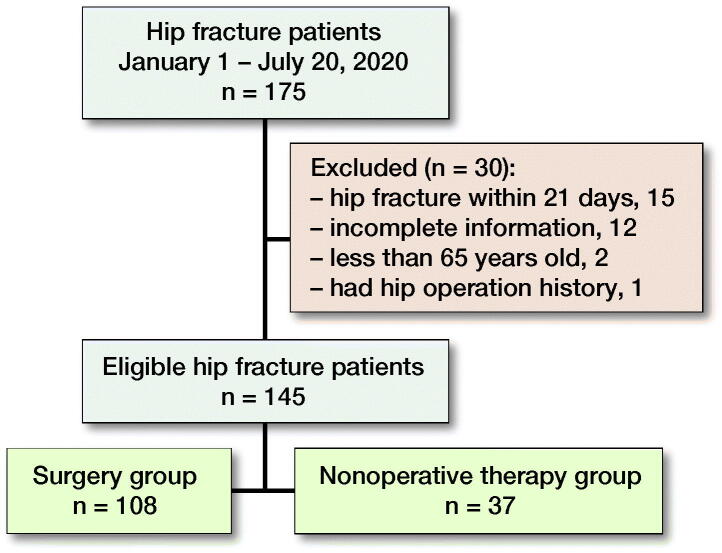
Flow chart of study design.

175 patients were initially identified (Figure 1). Finally, 145 patients were included, with 108 patients undergoing surgery and the remaining 37 patients receiving nonoperative treatment (Table 1). No patients were diagnosed with COVID-19 infection. Most patients were female. Hypertension and cerebral infarction were the most common comorbidities. We noted no statistically significant difference between the 2 groups in terms of comorbidities as well as prior medical history (smoking). The most common cause of fracture was simple fall (118, 81%) and most common type of fracture was femoral neck fracture (104, 72%). Only 5 patients suffered brain injury. Femoral neck fractures were more common in the surgery group (p < 0.001) and intertrochanteric fractures were more common in the nonoperative therapy group (p < 0.001). There was no statistically significant difference in traction treatment (p = 0.5) and analgesic treatment (p = 0.9) between the 2 groups. Antibiotic treatment was more common in the surgery group (p = 0.04) (Table 1).

**Table 1.** Demographics, clinical characteristics, and treatment of elderly hip fracture patients during the COVID-19 pandemic

	All patients	Surgery	Nonoperative therapy	
Factor	n = 145	n = 108	n = 37	p-value
Demographics				
Female sex	90	70	20	0.2
Age, mean	72	72	74	0.2
range	66–81	65–79	66–85	
Chronic comorbidity				
Hypertension	81	62	19	0.5
Diabetes	29	18	11	0.09
Cerebral infarction	40	29	11	0.7
Coronary heart disease	23	15	8	0.3
Osteoporosis	10	9	1	0.4
Prior medical history				
Smoking	20	15	5	1.0
Alcohol	16	12	4	1.0
Other clinical characteristics				
Simple fall	118	88	30	1.0
Traffic accident	15	11	4	1.0
Fall from height	6	4	2	1.0
Sprain	6	5	1	1.0
Femoral fracture type				
Neck	105	88	17	< 0.001
Intertrochanteric	36	17	19	< 0.001
Subtrochanteric	4	3	1	1.0
Brain injury	5	4	1	1.0
Treatment				
Traction	41	32	9	0.5
Analgesics	116	86	30	0.8
Antibiotics	68	56	12	0.04

Surgery, which included plate internal fixation, dynamic hip screw, proximal femoral nail antirotation, hip replacement, and hollow screw internal fixation, was selected based on type of fracture. Days from injury to surgery, surgery time, and intraoperative blood loss were noted (Table 2).

**Table 2.** Intraoperative data of 108 surgical patients

	All	Plate fixation	Dynamic hip screw	Proximal antirotation nail	Hip replacement	Hollow screw fixation
	n = 108	n = 12	n = 6	n = 15	n = 68	n = 7
Femoral fracture typ, n						
neck	88	7	5	2	67	7
intertrochanteric	17	5	0	11	1	0
subtrochanteric	3	0	1	2	0	0
Days from injury to surgery	33	32	45	29	37	23
IQR	24–48	25–58	26–71	22–44	25–49	21–38
Surgery time, minutes	90	133	95	90	80	90
IQR	60–120	60–173	80–110	60–135	60–120	40–120
Intraoperative blood loss, mL	200	150	225	200	200	50
IQR	100–300	100–400	118–425	100–400	120–300	30–100

Prior to discharge, the laboratory test results showed that the nonoperative therapy group displayed lower lymphocyte counts, lower hemoglobin, higher level of CRP, and higher level of D-dimer (Table 3, see Supplementary data).

At the 1-month follow-up, the surgery group showed a lower incidence of deep venous thrombosis and pulmonary infection. In terms of functional outcome, the surgery group had a lower VAS score (p < 0.001) and a higher Harris Hip Score (p = 0.04), showing a better early outcome than the nonoperative therapy group (Table 4).

**Table 4.** 1-month follow-up results of 145 elderly hip fracture patients during the pandemic of COVID-19

	All patients	Surgery	Nonoperative therapy	
Items	n = 145	n = 108	n = 37	p-value
Deep venous thrombosis, n	20	11	9	0.03
Pulmonary infection, n	24	14	10	0.03
VAS score (0–10)	3.0	3.0	5.0	< 0.001
IQR	2.0–4.0	1.3–4.0	3.0–6.5	
Harris Hip Score (0–100)	69	71	66	0.04
IQR	57–82	60–84	55–82	

**Figure 2.**
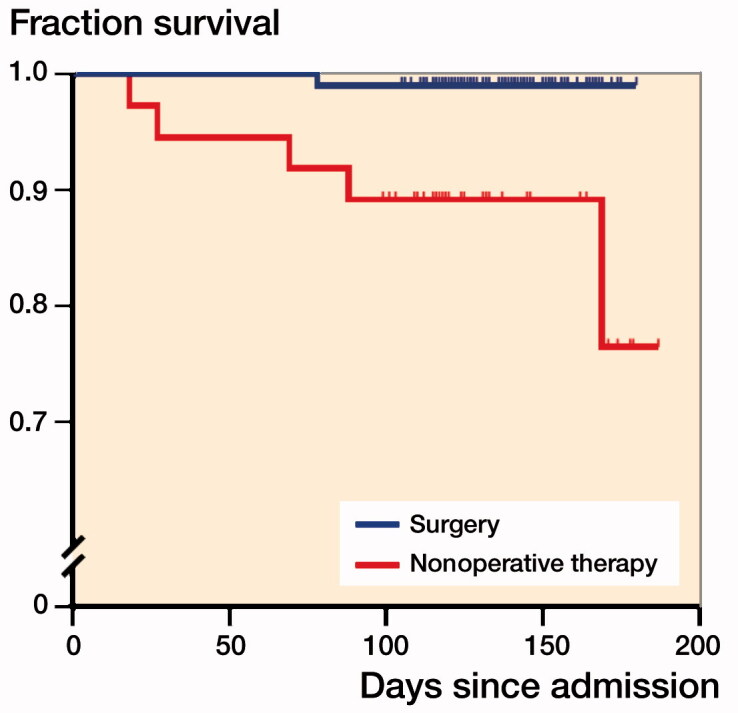
Kaplan–Meier curves of survival. Gehan–Breslow–Wilcoxon test p-value = 0.003; hazard ratio, surgery versus nonoperative therapy group = 0.06 (95% confidence interval 0.01–0.38).

At the 3-month follow-up, 9 patients from the surgery group and 3 patients from the nonoperative therapy group did not come to hospital for review or were unable to be contacted. Among the 133 remaining follow-up patients, 13/99 patients in the surgery group suffered from deep venous thrombosis, and 12/34 patients in the nonoperative therapy group (p = 0.004, Table 5). Pulmonary infection occurred in 15/99 patients in the surgery group and 16/34 patients in the nonoperative therapy group (p < 0.001). Patients in the surgery group showed a lower VAS score and better recovery of hip function than patients in the nonoperative group (p < 0.001 for VAS score; p = 0.04 for Harris Hip Score). Only 1 (1%) patient died in the surgery group, while 4 patients (12%) died in the nonoperative therapy group after 3-month follow-up (p = 0.02) (Table 6, Figure 2).

**Table 5.** 3-month follow-up results of 133 elderly hip fracture patients during the COVID-19 pandemic

	All patients	Surgery	Nonoperative therapy	
Items	n = 133	n = 99	n = 34	p-value ^a^
Deep venous thrombosis, n	25	13	12	0.004
Pulmonary infection, n	31	15	16	< 0.001
VAS score (0–10)	1.0	1.0	2.5	< 0.001
IQR	0.0–3.0	0.0–2.0	1.0–5.0	
Harris Hip Score (0–100)	71	73	70	0.04
IQR	63–85	66–86	58–84	
Dead	5	1	4	0.02

**^a^**Mann–Whitney U-test or chi-square test was selected to compare differences between surgery and conservative therapy where appropriate.

**Table 6.** Demographics, clinical characteristics, and causes of death of 6 dead elderly hip fracture patients during the COVID-19 pandemic

Patient	Therapy	Sex	Age	Hyper- tension	Dia- betes	Cerebral infarction	Other chronic comorbidity	Cause of death	Days from admission to death
1	surgical	female	84	+	+	+	Chronic obstructive pulmonary disease	Pulmonary infection	78
2	nonoperative	male	80	–	–	+	Pleural effusion, arrhythmia, atrial fibrillation	Heart failure	18
3	nonoperative	male	87	–	–	–	Pulmonary infection, pulmonary tuberculosis, pulmonary embolism	Pulmonary embolism	27
4	nonoperative	female	71	+	–	+	Coronary heart disease, arrhythmia	Heart failure	69
5	nonoperative	male	73	+	+	+	Coronary heart disease	Heart failure	88
6	nonoperative	male	85	+	+	–	Renal failure	Renal failure	169

## ^Discussion^

This study is the first to compare the outcomes of delayed surgery and nonoperative therapy for patients with hip fractures in the post-COVID-19 arena. This study includes 108 patients with delayed surgery and 37 patients with nonoperative therapy. Prior research has reported that the longer the delay in surgery, the higher the risk of poor outcomes and complications (Neuman et al. 2014). Most hip fracture patients are elderly with pulmonary dysfunction, meaning that pulmonary infection is more likely to occur when lying in bed (Sasabuchi et al. 2018). It is reported that as high as 6% of patients experience pulmonary infection during nonoperative management or delayed surgery (Goh et al. 2020). Therefore, prompt surgical repair of hip fractures is recommended. However, it is difficult to ensure all patients receive timely surgery due to various factors. Normal medical activity was disturbed by the outbreak of COVID-19. Although surgeons aimed to treat patients promptly, the limited medical resources resulted in surgical postponements. Such patients with hip fractures eventually crowded into hospitals post-COVID-19, while others chose nonoperative therapy. In our study, 13% of pulmonary infections were noted in the delayed surgery group, and 27% were noted in the nonoperative therapy group after 1-month follow-up. However, the percentage of pulmonary infection increased to 15% and 47% in the surgery group and nonoperative therapy group respectively after 3-month follow-up. Thus, although delayed surgery is not the optimal choice, it showed a decreased occurrence of pulmonary infection, possibly due to early mobilization and standing post-surgery.

Deep venous thrombosis is a common complication in patients with hip fractures. Although all patients are recommended prophylactic treatment including massage, muscle equal-length contraction, and passive joint movement, the odds of deep venous thrombosis (DVT) remain high. Previous studies have reported that the prevalence of preoperative DVT is 8% in hip fracture patients (Shin et al. 2016). In our study, 11 cases (10%) of DVT were seen in the surgery group and 9 cases (24%) of DVT were seen in the nonoperative therapy group after 1-month follow-up; the percentages were increased to 13% in the surgery group and 35% in the nonoperative therapy group after 3-month follow-up. Various reasons contribute to higher DVT rates, including aging, smoking, and surgery. Our results indicate that elevated D-dimer is 1 of the risk factors for venous thrombosis, consistent with previous studies (Xing et al. 2018). Thus, antithrombotic drugs were recommended for the patients in both the delayed surgery group and the nonoperative therapy group, especially for patients with elevated D-dimer levels (Forster and Stewart 2016). Treatments should be considered to avoid pulmonary embolus once deep venous thrombosis is noted (Spandorfer and Galanis 2015).

This study is limited by its small size and our conclusions may suffer from sample bias. With the extensive development of medical technology, delays in surgical treatment of hip fractures are rare. The COVID-19 outbreak has created a unique worldwide medical situation that has necessitated the delay of elective procedures. Our data strongly suggests that delayed surgery is superior to nonoperative therapy for hip fracture patients. With COVID-19 now under control in China, surgeons are now refocusing on delayed elective patients. As we did not collect data on physicians, possible variations in physician skill level may affect outcomes, especially in terms of complications. Although the 1-month mortality rate has been quoted to range from 3% to 24% post-surgery treatment and 31% post-conservative treatment (Prommik et al. 2019), no differences in death rates were observed between the two groups in our study after 1-month follow-up. However, 1 patient (1%) in the surgery group and 4 patients (12%) in the nonoperative therapy group were dead after 3-month follow-up. We report only on the short-term prognosis of hip fracture. The outcomes as regards malunion, non-union, infection, and mortality rate will be recorded in a future study. No data was collected on the cost for hip fracture patients. Requirement for nursing and rehabilitation care when staying in a nursing home, and various post-surgical complications including DVT, may increase the cost of treatment.

In summary, when elective procedures must be postponed, patients with hip fractures are more likely to benefit from delayed surgery rather than nonoperative therapy. Compared with nonoperative therapy, delayed surgery showed a lower likelihood of major complications. Furthermore, it decreased pain and resulted in increased mobilization and better function.

## Supplementary Material

Supplemental Material

## Supplementary data

Table 3 is available as supplementary data in the online version of this article, http://dx.doi.org/ 10.1080/17453674. 2020.1816617

## References

[CIT0001] Ekeloef S , Homilius M , Stilling M , Ekeloef P , Koyuncu S , Münster A B , Meyhoff C S , Gundel O , Holst-Knudsen J , Mathiesen O , Gögenur I . The effect of remote ischaemic preconditioning on myocardial injury in emergency hip fracture surgery (PIXIE trial): phase II randomised clinical trial. BMJ 2019; 367: 16395. 10.1136/bmj.l6395 PMC6891801 31801725

[CIT0002] Forster R , Stewart M . Anticoagulants (extended duration) for prevention of venous thromboembolism following total hip or knee replacement or hip fracture repair. Cochrane Database Syst Rev 2016; 3: CD004179. 27027384 10.1002/14651858.CD004179.pub2 PMC10332795

[CIT0003] Goh E L , Lerner R G , Achten J , Parsons N , Griffin X L , Costa P M L . Complications following hip fracture: results from the World Hip Trauma Evaluation cohort study. Injury 2020, 6: 1331–6. 32268962 10.1016/j.injury.2020.03.031 PMC7322551

[CIT0004] Neuman M D , Silber J H , Magaziner J S , Passarella M A , Mehta S , Werner R M . Survival and functional outcomes after hip fracture among nursing home residents. JAMA Intern Med 2014; 174: 1273–80. 25055155 10.1001/jamainternmed.2014.2362 PMC4122620

[CIT0005] NHCC. National Health Commission of China. [The State Council’s joint prevention and control mechanism for pneumonia epidemic in response to new coronavirus infection (6th edition).] Chinese. Accessed 2020 Mar 7. http://www.nhc.gov.cn/jkj/s3577/202003/4856d5b0458141fa9f376853224d41d7.shtml

[CIT0006] Papadimitriou N , Tsilidis K K , Orfanos P , Benetou V , Ntzani E E , Soerjomataram I , Künn-Nelen A , Pettersson-Kymmer U , Eriksson S , Brenner H , Schöttker B , Saum K U , Holleczek B , Grodstein F D , Feskanich D , Orsini N , Wolk A , Bellavia A , Wilsgaard T , Jørgensen L , Boffetta P , Trichopoulos D , Trichopoulou A . Burden of hip fracture using disability-adjusted life-years: a pooled analysis of prospective cohorts in the CHANCES consortium. Lancet Public Health 2017; 2: e239–e46. 29253489 10.1016/S2468-2667(17)30046-4

[CIT0007] Prommik P , Kolk H , Sarap P , Puuorg E , Harak E , Kukner A , Pääsuke M , Märtson A . Estonian hip fracture data from 2009 to 2017: high rates of non-operative management and high 1-year mortality. Acta Orthop 2019; 90: 159–64. 30669948 10.1080/17453674.2018.1562816 PMC6461069

[CIT0008] Sasabuchi Y , Matsui H , Lefor A K , Fushimi K , Yasunaga H . Timing of surgery for hip fractures in the elderly: a retrospective cohort study. Injury 2018; 49: 1848–54. 30097309 10.1016/j.injury.2018.07.026

[CIT0009] Shin W C , Woo S H , Lee S J , Lee J S , Kim C , Suh K T . Preoperative prevalence of and risk factors for venous thromboembolism in patients with a hip fracture: an indirect multidetector CT venography study. J Bone Joint Surg Am 2016; 98: 2089–95. 28002372 10.2106/JBJS.15.01329

[CIT0010] Sinvani L , Goldin M , Roofeh R , Idriss N , Goldman A , Klein Z , Mendelson D A , Carney M T . Implementation of hip fracture co-management program (AGS CoCare: Ortho®) in a large health system. J Am Geriatr Soc 2020; 389: 1519–27. 10.1111/jgs.16483 32391958

[CIT0011] Spandorfer J , Galanis T . Deep venous thrombosis. Ann Intern Med 2015; 162: ITC1. 10.7326/AITC201505050 25939012

[CIT0012] Xing F , Li L , Long Y , Xiang Z . Admission prevalence of deep vein thrombosis in elderly Chinese patients with hip fracture and a new predictor based on risk factors for thrombosis screening. BMC Musculoskelet Disord 2018; 19: 444. 30572863 10.1186/s12891-018-2371-5 PMC6302421

