# Supplementary material for: Delayed surgery versus nonoperative treatment for hip fractures in post-COVID-19 arena: a retrospective study of 145 patients
Source: Acta Orthop. 2020 Sep 8;91(6):639–43. doi: 10.1080/17453674.2020.1816617 (PMC8023940; doi:10.1080/17453674.2020.1816617)
Supplement: Supplemental Material [file IORT_A_1816617_SM3084.pdf]

Supplementary data

Table 3. Laboratory findings of elderly hip fracture patients prior to discharge during the COVID-19 pandemic. Values are median (IQR) **unless otherwise specified**

Items	Normal laboratory range	All patients n = 145	Surgery n = 108	Nonoperative therapy n = 37	p-value [a]
White blood cell count, $\times 10^9$/L	3.5–9.5	6.6 (5.5–8.1)	6.8 (5.5–8.0)	6.5 (5.4–8.3)	0.7
Lymphocyte count, $\times 10^9$/L	1.1–3.2	1.3 (1.0–1.8)	1.4 (1.0–1.8)	1.1(0.8–1.4)	0.007
Hemoglobin, mean (SD), g/L	130–175	113 (19)	116 (17)	106 (23)	0.003
ESR, mm/h	< 20	19 (12–40)	18 (11–39)	34 (15–46)	0.1
CRP, mg/L	< 5	12 (5.0–27)	11 (5.0–25)	32 (9.0–42)	0.008
D-dimer FEU [b]	< 0.5	1.6 (0.6–2.9)	1.4 (0.5–2.5)	2.4 (0.8–4.5)	0.05
TT, s	14–20	15 (14–17)	16 (14–17)	15 (14–17)	0.5
FIB, g/L	2.0–4.0	3.7 (3.1–4.3)	3.6 (3.0–4.2)	3.9 (3.1–4.7)	0.2
APTT, mean (SD), s	27–45	32 (7.2)	31 (6.2)	34 (9.5)	0.1
PT, s	11–16	12 (11–14)	12. (11–13)	12 (11–14)	0.4

[a] independent group t-test, Mann–Whitney U-test, chi-square test, or Fisher's exact test was selected to compare differences between surgery and conservative therapy where appropriate.
[b] FEU– fibrinogen equivalent units
